# The preoperative localisation of small parathyroid adenomas improves when adding Tc-99m-Sestamibi SPECT to multiphase contrast-enhanced CT

**DOI:** 10.1186/s13244-021-01016-3

**Published:** 2021-06-05

**Authors:** Patricia Sandqvist, Jacob Farnebo, Inga-Lena Nilsson, Per Grybäck, Anders Sundin, Alejandro Sanchez-Crespo

**Affiliations:** 1 Department of Molecular Medicine and Surgery, Karolinska Institute, Stockholm, Sweden; 2 Department of Oncology-Pathology, Karolinska Institute, SLL, 17177 Stockholm, Sweden; 3 Department of Medical Radiation Physics and Nuclear Medicine, Karolinska University Hospital, Stockholm, Sweden; 4 Department of Endocrine Tumours and Sarcoma, Karolinska University Hospital, Stockholm, Sweden; 5 Department of Surgical Sciences, Section for Radiology and Molecular Imaging, Uppsala University, Uppsala University Hospital, Uppsala, Sweden

**Keywords:** Parathyroid adenoma, Single-photon emission-computed tomography, 4D computed tomography, Parathyroidectomy, Contrast media

## Abstract

**Objectives:**

To investigate the incremental value of Sestamibi SPECT combined with a non-enhanced and contrast-enhanced CT, using SPECT/CT, for the preoperative localisation of small parathyroid adenomas (PTA).

**Methods:**

Retrospectively, 147 patients surgically cured from primary hyperparathyroidism, as verified by biochemistry 6 months postoperatively, were included. All patients had preoperatively undergone a dual time ^99m^Technetium-Sestamibi SPECT (S) with multiphase CT including native (N), arterial (A) and venous (V) phases. Independently, two radiologists blinded from both the surgical and the preoperative imaging reports, sequentially performed PTA localisation starting with either [A] or [V], thereafter [A + N] or [V + N] and finally with the complete [A + N + S] or [V + N + S]. PTA localisation was reported for each image-set. The readers results were combined and the diagnostic performance for each image set was determined. Sensitivity was also calculated for the different quartiles of PTA weight distribution.

**Results:**

The median adenoma weight was 315 mg. No statistically significant differences in diagnostic performance between arterial and venous based image sets were found. The net effect of adding [N] was to increase specificity. Sestamibi SPECT significantly increased the overall diagnostic accuracy for arterial- and venous-based image sets, *p* = 0.0008 and *p* = 0.001, respectively. [A + N + S] was found to have the highest diagnostic performance with 86.5% sensitivity and 94.9% overall accuracy. [A + N + S] was particularly advantageous for locating PTA in the lower weight quartiles.

**Conclusions:**

Native CT-phase and dual time point Sestamibi SPECT increase specificity and sensitivity, respectively. These, in combination with a single contrast-enhanced CT-phase is the most optimal examination protocol for preoperative localisation of PTA using SPECT/CT.

**Supplementary Information:**

The online version contains supplementary material available at 10.1186/s13244-021-01016-3.

## Key points

- Parathyroidectomy of increasingly lower weight adenomas challenges preoperative imaging based on SPECT/CT.
- The most optimal SPECT/CT imaging protocol is still under debate.
- For low weight adenomas, adding ^99m^Tc-Sestamibi SPECT to multiphase CT contributes to increase sensitivity.
- No difference in diagnostic performance was found between arterial and venous CT-phases.
- Including a native CT contributes to increase specificity

## Introduction

Preoperative localisation remains a challenge regarding small parathyroid adenomas (PTA). Ultrasound imaging and metastable Technetium-99 (Tc-99m)—Sestamibi SPECT with or without CT are the first-line imaging techniques for PTA localisation. PET is promising but has limited clinical availability. Improvements in PTA localisation with US generally rely on operator skills and the vendors’ development of, for instance, high-resolution transducers. On the other hand, PTA localisation based on SPECT/CT imaging additionally relies on the development and optimisation of image acquisition protocols with patient radiation doses as limiting factor.

In this regard, previous studies have compared the cumulative value of different combinations of Tc-99m-Sestamibi SPECT [S], native CT [N] and arterial [A] or venous [V] phase contrast-enhanced CT in a SPECT/CT imaging protocol. Sandqvist et al. 2017 showed in a retrospective study an increase in specificity when the image set [N] was added to [S] [1]. In a prospective clinical study, Sandqvist et al. 2019 reported a statistically significant increase in PTA localisation sensitivity when contrast-enhanced CT image sets [A + V] were added to [S + N] [2]. Andersen et al. 2018 found no differences in sensitivity between [S + N] and [S + N + V] [3]. Finally, Yeh et al. 2019 showed no differences in diagnostic performance after adding [S] to a [N + A + V] image protocol [4]. Furthermore, there is no general consensus on whether all, or merely one or two of the CT-phases are required for optimal image performance with regard to detection rate and radiation doses [5–7]. A common limitation occurring in many publications on this topic is the omission of the influence of the adenoma weight distribution on the results. Taken all these factors into account, this diagnostic landscape clearly calls for further analysis on the diagnostic value of each component in a multiphase contrast-enhanced Tc-99m-Sestamibi SPECT/CT image acquisition protocol for preoperative PTA localisation in relation to the adenoma weight.

The main goal of this work was to investigate the incremental value of adding [S] to [N + A + V] for PTA localisation based on a cohort of patients with well-defined adenoma weight. Secondary goals were first to study the role of [N] when combined with contrast-enhanced CT and finally elucidate whether both or only one of the contrast-enhanced phases are required.

## Material and methods

### Study population

**Fig. 1:**
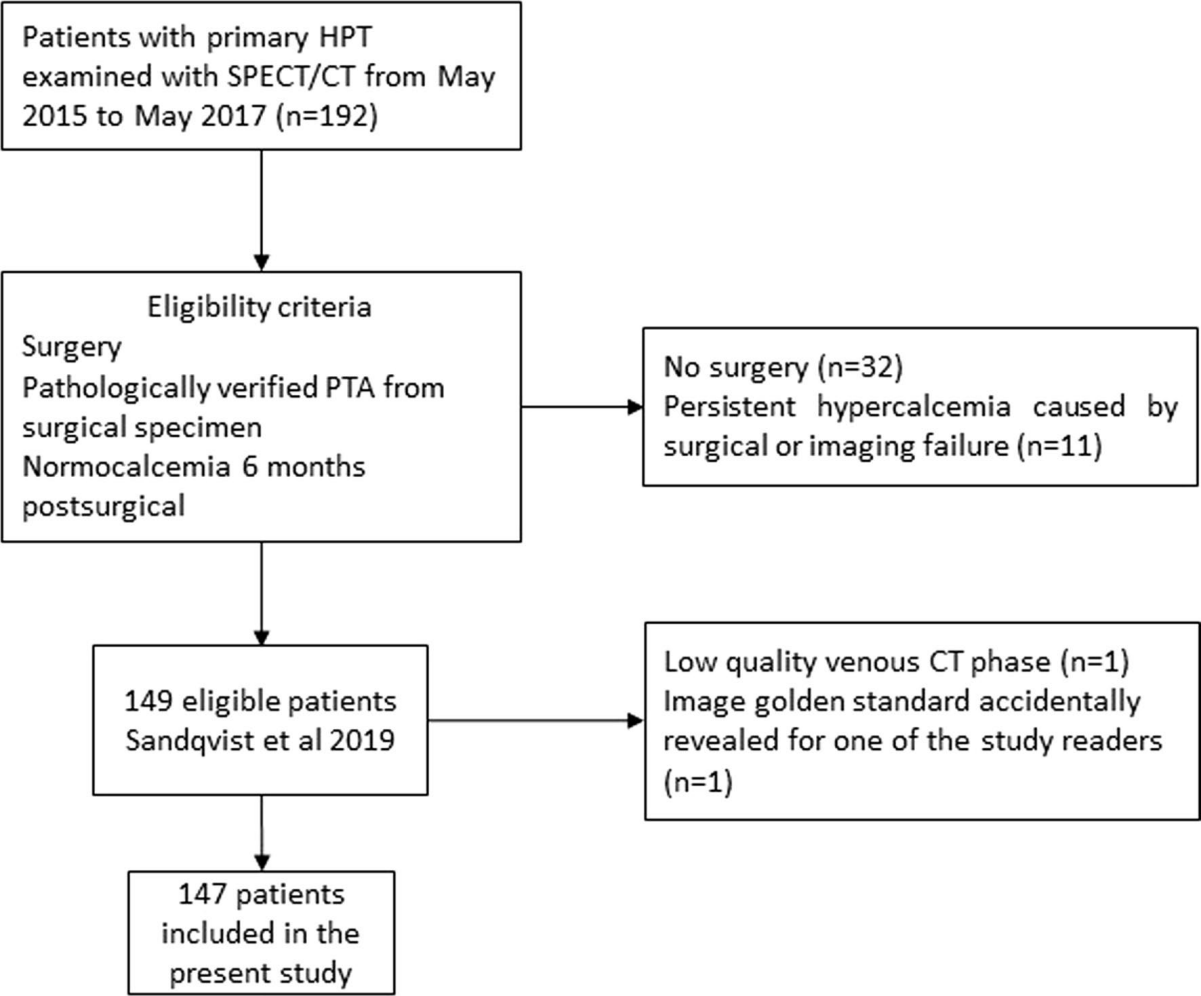
Flowchart of the study population

One hundred and forty-seven patients were retrospectively accrued from a prior study including 149 patients with primary hyperparathyroidism (pHPT) consecutively recruited between May 2015 and May 2017 at the Karolinska University Hospital, Stockholm [2], as shown in Fig. 1.

### SPECT/CT imaging acquisition

All patients underwent preoperatively a comprehensive diagnostic SPECT/CT examination in the supine position with their arms down. The following imaging acquisition sequence was used; 10 min after intravenous administration of 500 MBq of Tc-99m-Sestamibi (Polatom, Otwock, Poland), a native low-dose CT for attenuation correction was performed, followed by the first SPECT acquisition. 90 min after Tc-99m-Sestamibi administration a second SPECT acquisition was performed followed by a native CT with diagnostic quality. Thereafter approximately 1 mL/kg body weight + 10 ml of iodine-based contrast medium (Optiray 350 mg I/mL; Mallinckrodt, Raleigh, NC) was administered at 3 to 3.5 mL/s by means of a power injector (Medrad Stellant; Bayer Healthcare, Whippany, NJ). Arterial and venous contrast-enhanced CT acquisitions were performed with 35- and 75-s delay, respectively. All SPECT/CT examinations were performed on a dual-head Siemens Symbia T16 SPECT/CT scanner (Siemens Healthcare, Erlangen, Germany) equipped with ultra-high-resolution collimators. SPECT acquisition parameters were 180-degree rotation, 64 angles, 20 s/angle and 128 × 128 image matrix size. SPECT images were corrected for scattering and attenuation using the native CT examination and subsequently reconstructed with a standard OSEM algorithm. Diagnostic CT imaging were acquired at 130 kV, 40 mAs, dose modulated tube current with 0.75 mm collimation, 40 cm scan length with large transaxial field of view (FOV) for native phase (to match the SPECT FOV for attenuation correction) and 25 cm scan length with small transaxial FOV (to optimise the resolution) for contrast-enhanced CT.

### Preparation of ground truth for PTA localisation

For comparisons through this study, the true localisation of the PTA, for each patient was marked in a separate CT image, the ground truth (GT) image. In order to produce a GT as close as possible to the true location of the PTA in the SPECT/CT images, an experienced radiologist and nuclear medicine specialist (P.S.), with more than 15 years of experience, retrospectively correlated PTA localisation based on all available imaging information [S + N + A + V] with the PTA location derived from the surgical report and verified by the histopathological examination of the surgical specimen, which also included PTA weight. The latter was also used to correlate with the PTA size measured on CT.

### Incremental SPECT/CT analysis for PTA localisation

Two radiologists with long clinical nuclear medicine experience (J.F. and A.S.) and blinded from patient identity and the CT images containing the GT were instructed to independently perform PTA localisation based on an incremental image sequence. The use of two radiologists, limited the number of different combinations of images sets that could incrementally be used without biasing the readers. Hence, and in accordance with the study aims, each reader performed PTA localisation using the following image set sequence, [A or V] followed by [(A or V) + N] and finally [(A or V) + N + S].

**Fig. 2:**
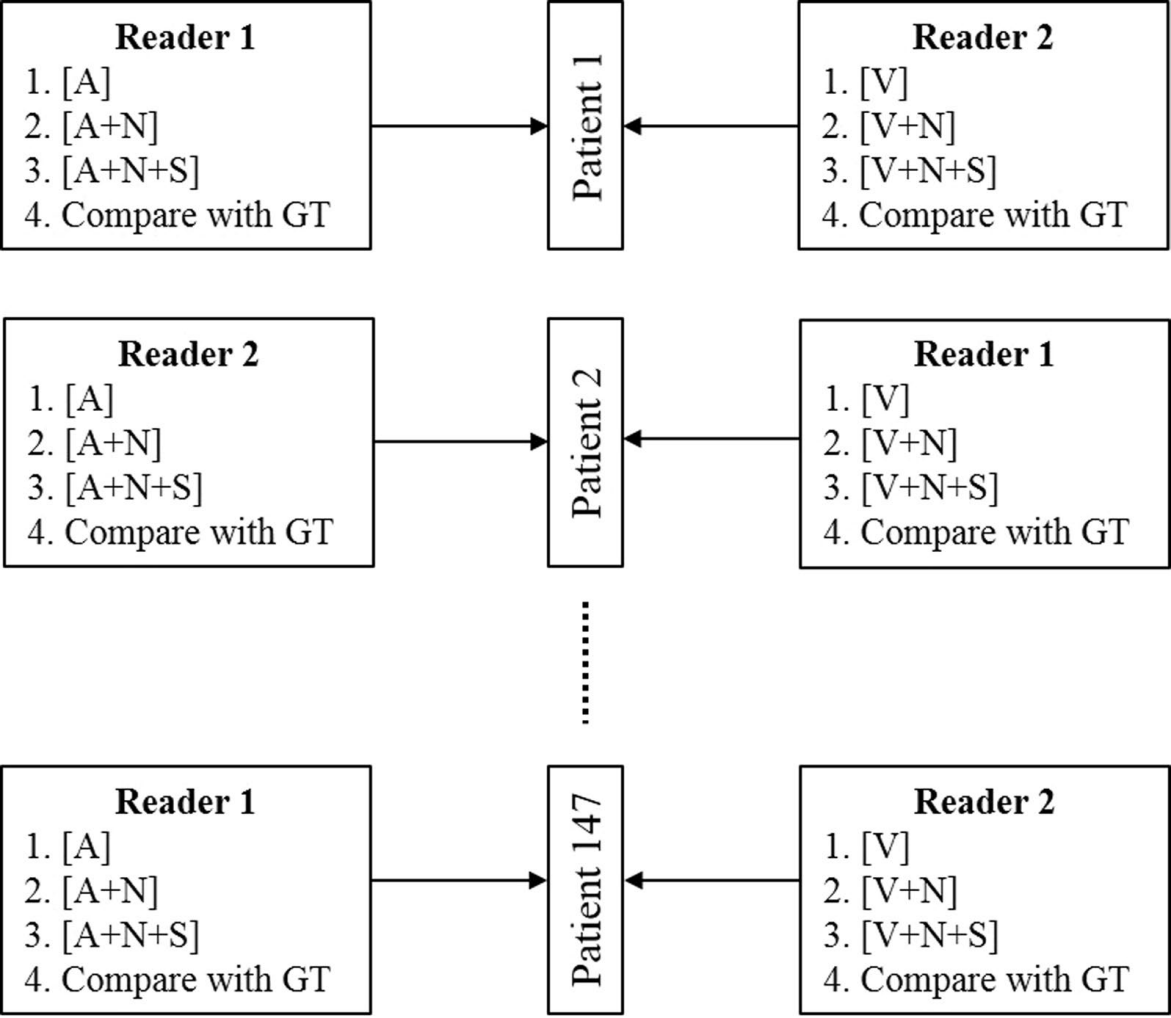
Study design and image set analysis sequence

Additionally, to investigate differences between the [A] and the [V] image sets, each reader alternated between arterial- and venous-based image sets every other patient, as described in Fig. 2. In the final step of the analysis, each radiologist compared their PTA localisation at each step of the image set sequence with the GT and documented the results.

### Statistical methods

Sensitivity, specificity and overall diagnostic accuracy were calculated for each image set. For these values, 95% confidence intervals (CI) were calculated using the binomial Clopper–Pearson exact method. For the calculation of specificity and accuracy we assumed that all patients had 4 parathyroid glands. Absolute differences (with 95% CI) in both PTA localisation sensitivity, specificity and accuracy between different image sets were calculated and the two-sided McNemar’s test for matched pairs with Yates’ correction was used to test the null hypothesis of no differences between image sets.

Data analysis and statistical testing were performed using both MATLAB (MathWorks, version R2019a) and R (version 3.6.3). All statistical tests were two-sided with a significance level at 0.05.

## Results

**Fig. 3:**
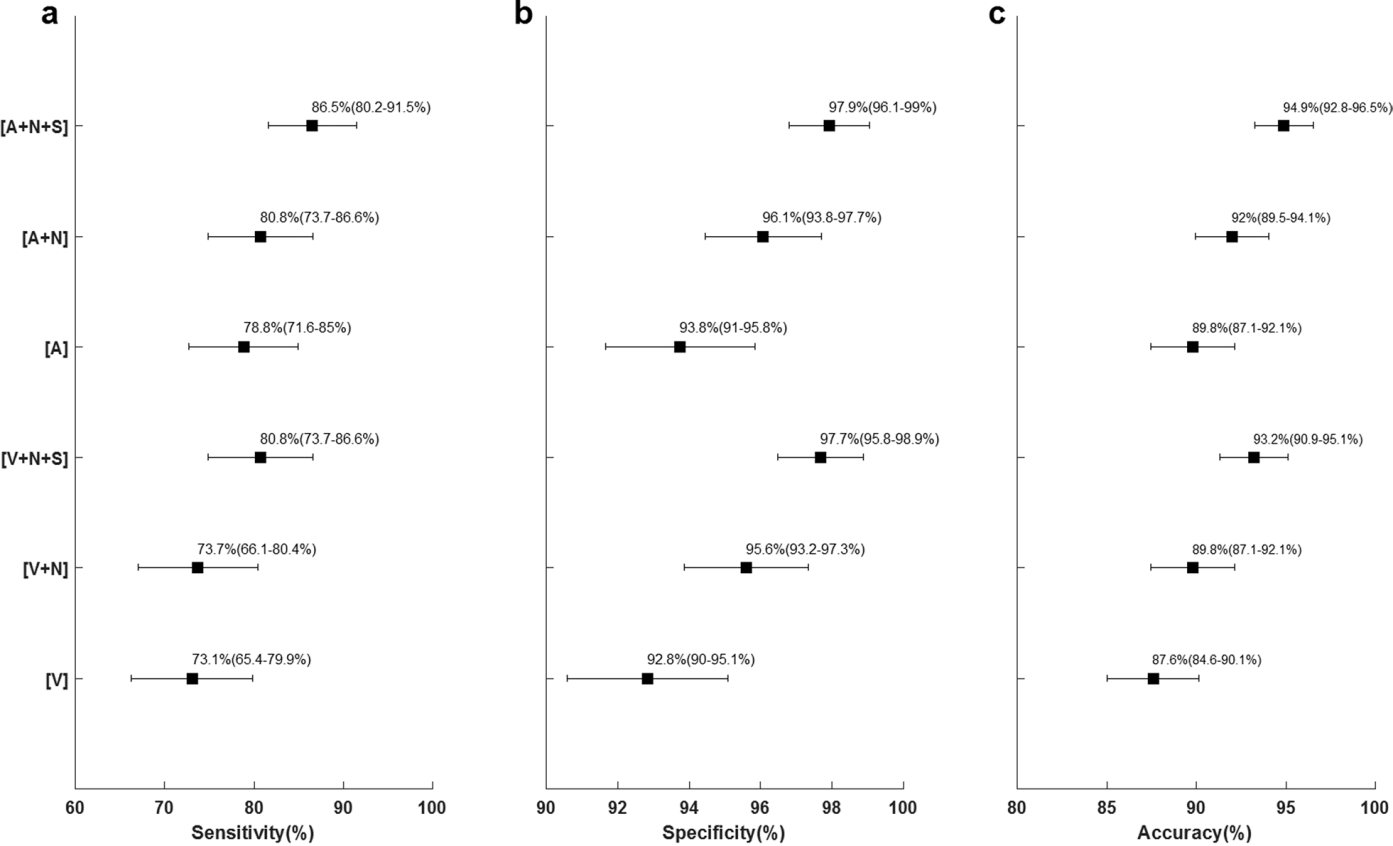
Diagnostic sensitivity (**a**), specificity (**b**) and overall diagnostic accuracy (**c**), with 95% confidence intervals, for parathyroid adenoma localisation of the individual image sets

The patients’ characteristics are summarised in Table 1. This cohort comprised a total of 147 patients (110 women and 37 men), harbouring in total 156 PTA with median weight 315 mg. Eight of these patients had multiglandular disease (1 patient had 3 adenomas and 7 patients had 2 adenomas). In this cohort, the adenomas (GT) were distributed in the following quadrants: left superior *n* = 40, left inferior *n* = 36, right superior *n* = 26, right inferior *n* = 49. Five adenomas were found in ectopic locations (Intrathyroidal, Mediastinum, Carotid sheath). Sensitivity, specificity and overall diagnostic accuracy increased when more image sets were available for the reader (Fig. 3).

**Table 1:** Patient characteristics

	All patients (*n* = 147)	Men (*n* = 37)	Women (*n* = 110)
Age (years)	62.2 ± 12.3	58.7 ± 11.8	63.4 ± 12.3
BMI	25.8 ± 4.7	27.0 ± 2.6	25.4 ± 5.2
Operative PTH (pmol/L)	10.8 ± 4.6	11.9 ± 5.0	10.4 ± 4.4
Postoperative PTH (pmol/L)	2.3 ± 2.0	2.2 ± 2.3	2.3 ± 2.0
Preoperative ionised Ca (mmol/L)	1.44 ± 0.08	1.45 ± 0.08	1.43 ± 0.08
Postoperative ionised Ca (mmol/L)	1.27 ± 0.06	1.26 ± 0.06	1.27 ± 0.05
Median adenoma weight (mg)	315 (405)	388 (754)	300 (323)

If not specified, values are mean plus minus standard deviation. Data in parentheses are interquartile ranges

*BMI* body mass index, *PTH* parathyroid hormone, *Ca* calcium

Adding [S] to [A + N] resulted in 9 more adenomas correctly located (out of 156) without excluding any true positive initially found by [A + N]. Adding [S] to [V + N] resulted in 14 additional correctly localised adenomas. Three true positive adenomas in [V + N] were incorrectly deemed as false positive after adding [S] (Additional file 1: Supplementary Table 1). Statistically, as shown in Fig. 3, conventional CT imaging alone had lower sensitivity, specificity and accuracy with wider 95% CI compared to combined Sestamibi SPECT/CT. Figure 3 also reveals that the combined [A + N + S] image set reached the highest overall diagnostic performance with an accuracy of 94.9% (95% CI, 92.8–96.5) and [V] the lowest, 87.6 (95% CI 84.6–90.1), difference 7.3% (*p* = 0.000003).

**Fig. 4:**
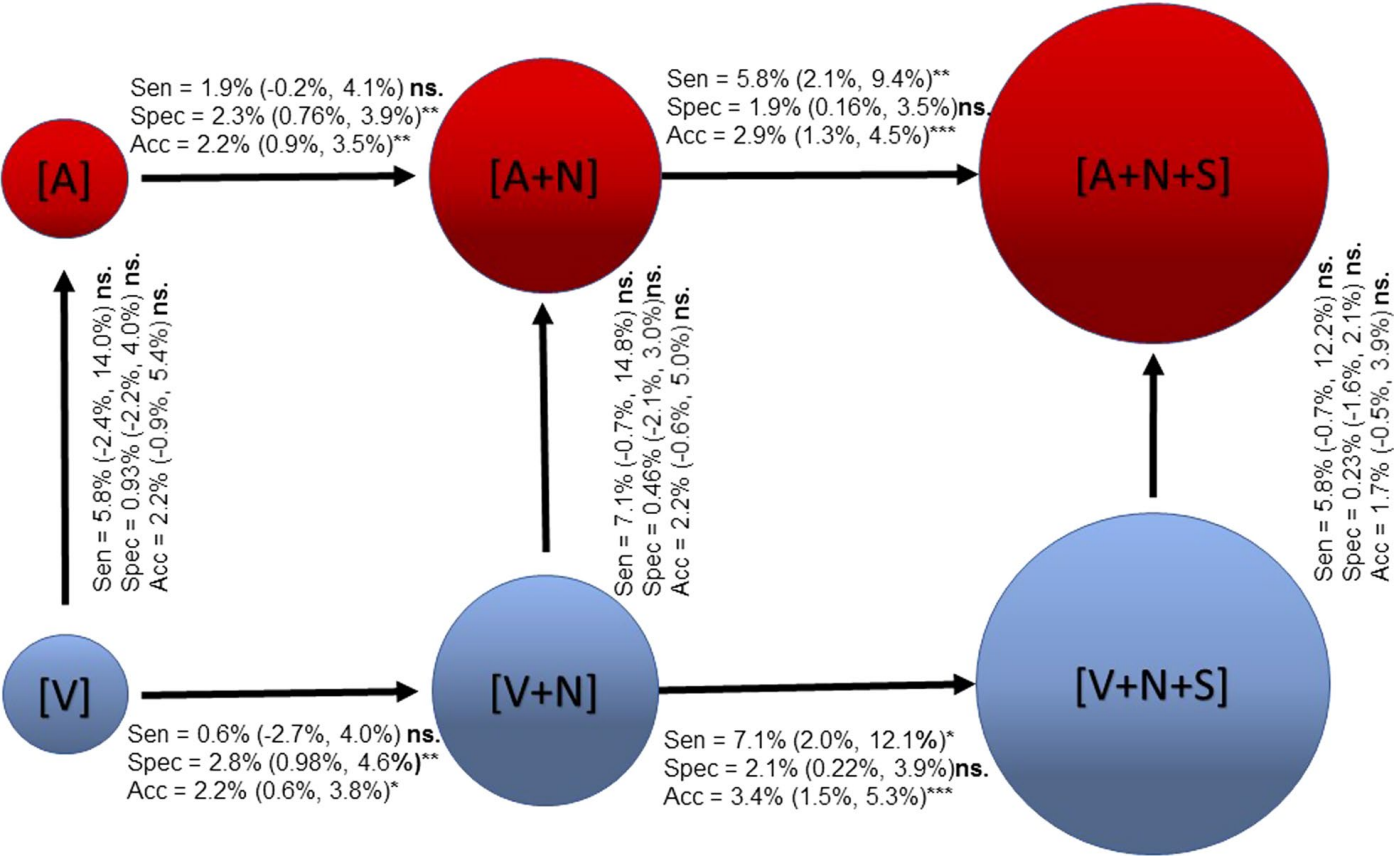
Absolute difference in diagnostic sensitivity, specificity and overall accuracy when adding image sets (horizontal direction of the arrows) and when comparing the venous- and the arterial-based image sets (vertical direction of the arrows). In parenthesis 95% confidence interval. ns = non-significant; (*) *p* ≤ 0.05; (**) *p* ≤ 0.01; (***) *p* ≤ 0.0001

No change in PTA localisation sensitivity and overall diagnostic accuracy between arterial and venous phase-based image sets was observed (Fig. 4). Further, adding native CT to a contrast-enhanced CT increased specificity and consequently improved overall accuracy for both arterial (*p* = 0.0019) and venous (*p* = 0.0123) phase-based image sets (Additional file 1: Supplementary Table 2). Figure 4 also reveals that the combined [A + N + S] image set resulted in a 2.9% absolute increase in diagnostic accuracy for correct PTA localisation than [A + N] alone (*p* = 0.0008). Likewise, the [V + N + S] image set showed 3.4% absolute increase in diagnostic accuracy than [V + N] alone (*p* = 0.001).

**Fig. 5:**
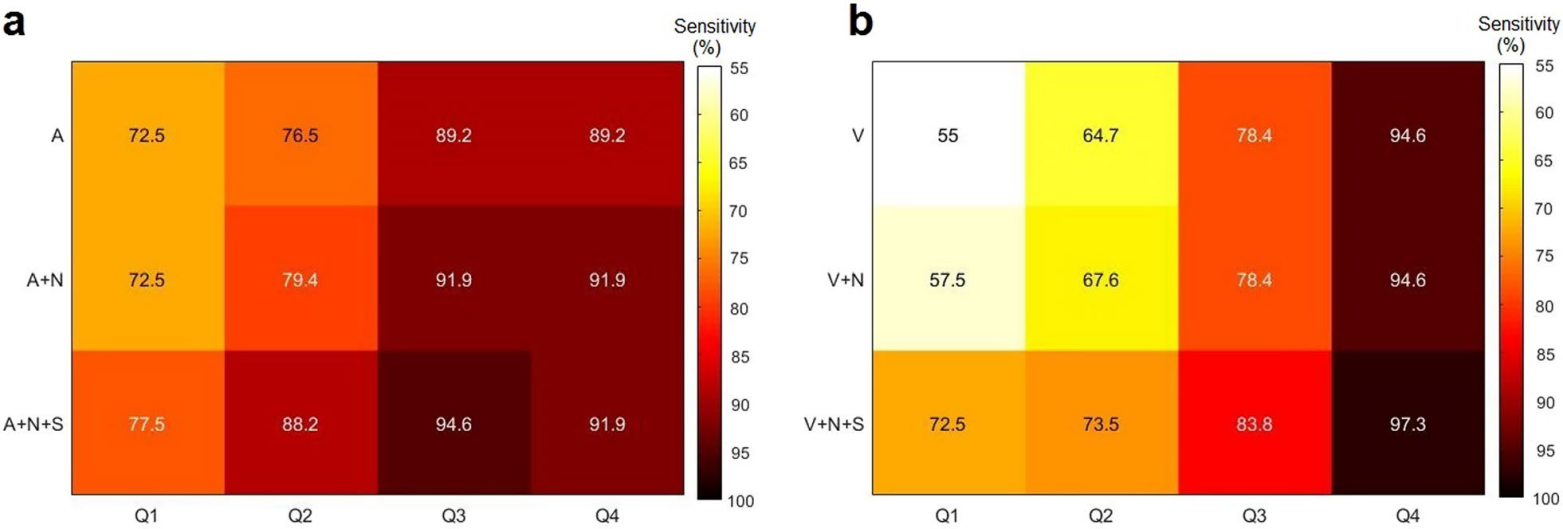
Sensitivity heatmap for parathyroid adenoma localisation based on either arterial-based image sets (**a**) or venous-based image sets (**b**) for different adenoma weight quartiles (upper limits for Q1, Q2 and Q3 are 200 mg, 315 mg and 609 mg, respectively)

Figure 5 reveals that the PTA weight distribution has a profound effect on the sensitivity for both arterial- (Fig. 5a) and venous-based image sets (Fig. 5b). Additionally, Fig. 5 also shows that sensitivity increased along with incremental image components up to Q3, but only minor differences were observed in Q4 (> 609 mg). As an example, Fig. 5b shows a pronounce sensitivity gain from 57.5% to 72.5% after adding Sestamibi SPECT [S] to [V + N] for Q1.

**Fig. 6:**
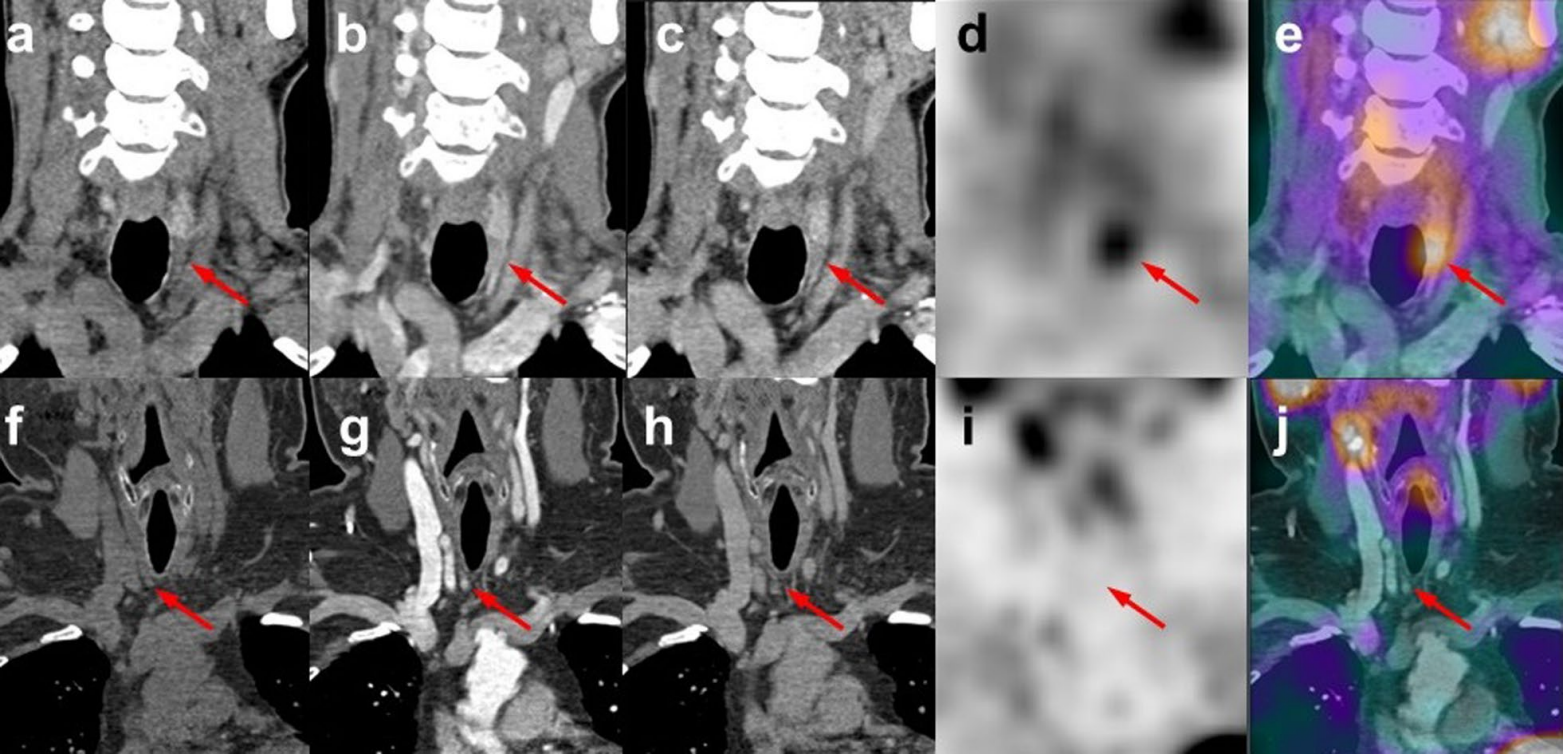
Multiphase SPECT/CT coronal views for 2 patients, upper and lower rows, respectively. Native phase (**a** and **f**), arterial phase (**b** and **g**), venous phase (**c** and **h**) and 90 min Tc-99m-Sestamibi SPECT (**d** and **i**) and fused SPECT and arterial CT phase (**e** and **j**). Arrows point to the anatomical localisation of the parathyroid adenomas with a weight of 222 mg and 205 mg for the upper and the lower rows, respectively

Figure 6 shows the PTA localisation for two patients, as an example of the complementary diagnostic value for different SPECT/CT image sets. For the patient in the upper row, SPECT shows a distinct focal Sestamibi uptake in a region where the CT findings were not conclusive. Of notice, as revealed in Fig. 6 b, the arterial contrast bolus for this patient was not optimally timed, reflecting the variability in the [A] quality between patients, for example due to differences in cardiovascular circulation. On the contrary, for the patient shown in the lower row, there is an evident contrast enhancing PTA but without Sestamibi uptake.

## Discussion

The results from this study indicate that compared to conventional CT imaging alone (based on either arterial or venous image sets), Sestamibi SPECT adds valuable information that increases the overall diagnostic performance for preoperative PTA localisation, especially for small adenomas. This is of clinical importance since earlier biochemical diagnosis together with a widening indication for surgery has resulted in a clear trend towards surgical removal of adenomas with increasingly lower weights.

Almquist M et al. 2010 have shown a decrease in median PTA weight from 725 to 560 mg between 1990 and 2007 [8] and McCoy et al. 2014, a decrease in average PTA weight from 1214 mg till 909 mg during the period 1997 to 2011 [9]. Furthermore, a recent population-based study, with 4890 histologically confirmed PTA, from a European surgical registry for endocrine tumours [10] showed a median PTA weight of 940 mg (interquartile range (IQR) 1570 mg). Compared to these results, our cohort was dominated by patients with small weight adenomas [median 315 mg (IQR 405)]. The profound incremental value of adding Sestamibi SPECT to [A/V + N] is clearly coupled to this group of adenoma weights and is less important for larger adenomas (Figs. 4, 5). This finding contradicts previously published results by Yeh et al. [4], which showed similar sensitivity for [A + V + N] and [A + V + N + S]. Since the patient characteristics of the cohort studied by Yeh et al., with regard to adenoma weight distribution and serum concentration of ionised calcium were not reported, possible differences of those parameters between cohorts could be a reason for this discrepancy. Further there was a clear difference in the incidence of multiglandular disease between cohorts (19.8% in Yeh et al., 5.4% in this study). As previously shown, multiglandularity results in lower sensitivity for Sestamibi SPECT alone [1]. In addition to PTA weight, there are other factors like the anatomical position of the PTA and the occurrence of goiter that could also influence the localisation sensitivity.

Figure 4 demonstrates that by adding a native CT phase to the [A] or [V] based image sets, a highly significant increase in PTA localisation specificity is achieved. This could be explained by the generally lower attenuation in the native CT-phase of PTA compared to the normal thyroid, mean 47 and 77 Hounsfield units, respectively [2]. Whether this increase in specificity is also valid when [N] is added to an image set containing a contrast-enhanced CT phase combined with Sestamibi SPECT, [A + S] or [V + S], could not be proven, since these image sets were not possible to include for review in our study design without introducing reader bias in the results.

PTA localisation sensitivity was statistically similar for arterial and venous phase-based image sets, suggesting a redundancy in the simultaneous use of two different contrast-enhanced CT phases. However, we found that a total of 18 adenomas seen in the [A + N + S] were unnoticed in the [V + N + S] image set, but vice versa only 9 extra adenomas were revealed (Additional file 1: Supplementary Table 1). Hence, in view of these results and in line with the international regulations of the clinical use of ionising radiation, we have at our institution excluded the venous phase, resulting in a 20% reduction in total radiation dose.

Remarkably, considering the small sized PTA in our cohort (median 315 mg), the obtained sensitivity for [A + N + S] was still comparable to that reported for PET/CT with F-18-fluorocholine (about 94% sensitivity) [11] and with C-11-methionine (sensitivity ranging from 69 to 74%) [12, 13]. Of notice, in the study by Beheshti et al., the median adenoma weight was as large as 1000 mg [11] which clearly can explain the slightly better results. The study by Weber T et al. 2016 reported a mean adenoma weight of 580 mg (589 mg in our cohort) [9] and the study by Kluijfhout WP et al. 2016 did not report adenoma weights [13].

Worldwide, the availability of SPECT/CT is considerably higher than that of PET/CT. Furthermore, the availability of F-18-fluorocholine or C-11-methionine is generally more limited than that of generator produced Tc-99m-Sestamibi. Hence, we believe that Sestamibi SPECT/CT, still for many years, will continue to constitute the mainstay in nuclear medicine imaging for preoperative localisation of PTA.

### Limitations of the study

This study is based on only two experienced readers reviewing different contrast-enhanced CT image sets for each patient and, as Fig. 2 describes, switching for every other patient. This methodology does not allow for an inter-observer agreement analysis, limiting the study to comparing the combined findings from these independent readers against a GT. For each patient, an experienced radiologist (P.S.) retrospectively determined the GT by connecting the pathological report (identifying which of the surgical specimens is the PTA) with the surgical report (describing where this PTA was removed in relation to anatomical landmarks like the thyroid and head neck quadrants) and the PTA anatomical location as seen in the complete radiological examination. Further, the radiological dimensions of the defined GT were matched against the weight of the confirmed adenoma, as another quality measure. In every link of this chain there are possibilities for misinterpretations, which may lead to an erroneous GT. However, this is a common problem to all studies published on this topic, including those using only anatomical quadrant-based comparisons. The method used in our study is, to our knowledge, the most thorough way to couple the surgical and radiological findings.

## Conclusions

In conclusion, the highest diagnostic performance in localisation of low weight PTA was achieved using a dual time 99mTc-Sestamibi SPECT/CT imaging protocol including a native CT and an arterial contrast-enhanced CT phase.

## Supplementary Information

**Additional file 1: Supplementary Table 1**. Overall study results for each image set, including number of total adenoma sites, total number of true positives, true negatives, false positives, false negatives, sensitivity, specificity, accuracy. **Supplementary Table 2**. Inference testing between image sets with regards to diagnostic performance. McNemars test was used with a significance level set to 0.05. n.s. = not significant.

## Data Availability

The anonymised datasets used and/or analysed during the current study are available from the corresponding author on reasonable request. In the supplementary material, we provide with the complete sampled data for each individual image set.

## Abbreviations

A: Arterial phase
GT: Ground truth
N: Native phase
S: Tc-99m-Sestamibi SPECT
V: Venous phase
BMI: Body mass index
Ca: Calcium
CI: Confidence interval
pHPT: Primary hyperparathyroidism
PTA: Parathyroid adenoma
PTH: Parathyroid hormone
Q: Quartiles
Tc-99m: Metastable Technetium-99
