# Supplementary material for: The preoperative localisation of small parathyroid adenomas improves when adding Tc-99m-Sestamibi SPECT to multiphase contrast-enhanced CT
Source: Insights Imaging. 2021 Jun 5;12:72. doi: 10.1186/s13244-021-01016-3 (PMC8179871; doi:10.1186/s13244-021-01016-3)
Supplement: Supplementary file 1 — Additional file 1: Supplementary Table 1. Overall study results for each image set, including number of total adenoma sites, total number of true positives, true negatives, false positives, false negatives, sensitivity, specificity, accuracy. Supplementary Table 2. Inference testing between image sets with regards to diagnostic performance. McNemars test was used with a significance level set to 0.05. n.s. = not significant. [file 13244_2021_1016_MOESM1_ESM.docx]

**ELECTRONIC SUPPLEMENTARY MATERIAL**

|  | **[A]** | **[A+N]** | **[A+N+S]** | **[V]** | **[V+N]** | **[V+N+S]** |
| --- | --- | --- | --- | --- | --- | --- |
| **Patients** | 147 | 147 | 147 | 147 | 147 | 147 |
| **Adenomas** | 156 | 156 | 156 | 156 | 156 | 156 |
| **Number of sites** | 588 | 588 | 588 | 588 | 588 | 588 |
| **TP** | 123 | 126 | 135 | 114 | 115 | 126 |
| **FN** | 33 | 30 | 21 | 42 | 41 | 30 |
| **FP** | 27 | 17 | 9 | 31 | 19 | 10 |
| **TN** | 405 | 415 | 423 | 401 | 413 | 422 |
| **Sensitivity (%)** | 78,8 | 80,8 | 86,5 | 73,1 | 73,7 | 80,8 |
| **Specificity (%)** | 93,8 | 96,1 | 97,9 | 92,8 | 95,6 | 97,7 |
| **Accuracy (%)** | 89,8 | 92,0 | 94,9 | 87,6 | 89,8 | 93,2 |

Table 1. Overall study results for each image set. TP= True positive; FN= False negative; FP= False positive; TN=True negative.

|  | **Sensitivity** | **Specificity** | **Accuracy** |
| --- | --- | --- | --- |
| **[A] vs [A+N]** | n.s. | 0.009 | 0.002 |
| **[A+N] vs [A+N+S]** | 0.008 | n.s. | 0.001 |
| **[A] vs [A+N+S]** | 0.001 | 0.001 | 0.000001 |
| **[V] vs [V+N]** | n.s. | 0.006 | 0.012 |
| **[V+N] vs [V+N+S]** | 0.015 | n.s. | 0.001 |
| **[V] vs [V+N+S]** | 0.025 | 0.001 | 0.000007 |
| **[A] vs [V]** | n.s. | n.s. | n.s. |
| **[A+N] vs [V+N]** | n.s. | n.s. | n.s. |
| **[A+N+S] vs [V+N+S]** | n.s. | n.s | n.s. |

Table 2. Inference testing using McNemars test, p-values >0.05 are considered not significant (n.s.).
